# Is Cold Storage Still a Must? Evaluating Room‐Temperature OnabotulinumtoxinA for Glabellar Lines Treatment

**DOI:** 10.1111/jocd.70554

**Published:** 2025-11-18

**Authors:** Ruth Yimna Del Socorro Blanco Rodriguez, Giancarlo De La Torre Canales, César Nunes Giracca, Thaís Caroline Schwartz Saldanha Vaz, Gislaine Paulovski, Lucas Alexandre de Sousa Mendes, Jairo Matozinho Cordeiro, Fábio Seigi Murakami, Mariana Barbosa Câmara‐Souza

**Affiliations:** ^1^ Ingá University Center ‐ UNINGÁ Maringá Paraná Brazil; ^2^ Egas Moniz Center for Interdisciplinary Research (CiiEM) Egas Moniz School of Health and Science Almada Portugal; ^3^ Division of Oral Rehabilitation, Department of Dental Medicine Karolinska Institutet Stockholm Sweden; ^4^ Federal University of Santa Catarina Florianópolis Santa Catarina Brazil; ^5^ Aesthetic Research Laboratory, Postgraduate Program in Pharmaceutical Sciences, Department of Pharmacy Federal University of Paraná Curitiba Paraná Brazil; ^6^ University Center of Associated Teaching Faculties São João da Boa Vista São Paulo Brazil; ^7^ Araras Dental School Hermínio Ometto Foundation Araras São Paulo Brazil

**Keywords:** botulinum toxins type A, reconstitution, temperature

## Abstract

**Background:**

New botulinum toxin type A (BoNT‐A) formulations eliminated cold‐chain storage before reconstitution. Nonetheless, concerns remain about BoNT‐A properties when stored at room temperature after reconstitution.

**Aim:**

This study aimed to evaluate the sterility and effectiveness of BoNT‐A storage at room temperature in glabellar wrinkles.

**Methods:**

BoNT‐A (Allergan Botox, Brazil) was reconstituted and maintained in a stove at 37°C for 60 days and then tested for sterility according to the Pharmacopeia guidelines. Then, sixty patients aged 25–60 years were divided into two groups: Group 1 (G1) received BoNT‐A immediately after reconstitution, and Group 2 (G2) received BoNT‐A after 7 days at room temperature. Participants were assessed for wrinkle severity using the Merz Scale, satisfaction with treatment using the Face‐Q scale, and muscle activity by electromyography. Data were collected before injections and after 15, 30, 60, and 90 days.

**Results:**

Absence of turbidity evidenced no microbial growth in all periods analyzed. Both groups showed significant reductions in glabellar contraction (*p* < 0.001), wrinkle severity (*p* < 0.05), and improved satisfaction. G1 presented lower wrinkle severity, while G2 reported higher satisfaction (*p* < 0.05).

**Conclusion:**

Room temperature and longer storage periods may not affect OnabotulinumtoxinA sterility and clinical results.

## 1 Introduction

Over the past two decades, facial aesthetic medicine has experienced remarkable growth, particularly with the widespread adoption of neuromodulators. Among these, botulinum toxin type A (BoNT‐A) has seen near‐exponential growth in clinical use, driven by its expanding indications across both medical and cosmetic fields—especially for the treatment of dynamic facial expression lines [1]. According to the 2024 International Society of Aesthetic Plastic Surgery (ISAPS) global survey on aesthetic procedures, the use of BoNT‐A increased by 26.9% between 2020 and 2024, making it the most performed non‐surgical aesthetic intervention worldwide [2].

The clinical efficacy of BoNT‐A in aesthetic applications is influenced by several factors, including wrinkle severity, injection technique, dosage, reconstitution practices, storage conditions, and the time elapsed between reconstitution and administration [3]. Different commercial brands provide varying storage guidelines for their products, with recommendations ranging from refrigeration to freezing prior to reconstitution. Additionally, most manufacturers advise using the reconstituted toxin within 24–72 h, which can present logistical challenges in clinical practice [4, 5].

However, expert consensus indicates that BoNT‐A may be safely refrigerated or even refrozen for up to 4 weeks post‐reconstitution without compromising clinical efficacy or sterility [6]. Supporting this, several studies have demonstrated sustained effectiveness of BoNT‐A in the treatment of glabellar, periorbital, and forehead rhytids even after 6 weeks—or in some cases, 6 months—of refrigerated or frozen storage following reconstitution [7]. Moreover, extended post‐reconstitution storage for several weeks or months does not appear to increase the risk of bacterial or fungal contamination [8, 9].

The emergence of second and third‐generation BoNT‐A formulations, such as incobotulinumtoxinA and daxxibotulinumtoxinA, has further advanced the field by eliminating the need for cold‐chain storage before reconstitution. These formulations are stable at room temperature (≤ 25°C) for up to 36 months while maintaining clinical efficacy for aesthetic indications [10, 11]. Nonetheless, concerns remain regarding the stability, sterility, and potency of BoNT‐A products when stored at room temperature after reconstitution. Animal studies suggest that storage at varying temperatures, including room temperature (20°C ± 5°C), does not significantly affect BoNT‐A's potency or safety for up to 48 weeks [12]. However, no clinical studies to date have evaluated the efficacy, durability, or microbiological safety of BoNT‐A after extended room temperature storage post‐reconstitution—particularly in tropical countries that rely on imported formulations.

Therefore, the present study aims to assess the sterility and clinical efficacy of onabotulinumtoxinA stored at room temperature for extended periods following reconstitution, in the treatment of dynamic glabellar lines, compared to freshly reconstituted toxin.

## 2 Material and Methods

### 2.1 Experimental Design

This research comprised two phases. In the first one, BoNT‐A (Allergan Botox, Brazil) was tested for sterility after long‐term room temperature storage according to the Brazilian Pharmacopeia guidelines. The second phase consisted of a randomized controlled single‐blinded study, approved by the Research Ethics Committee of Uningá University, Paraná, Brazil (CAAE #59261322.0.0000.5220), assessing the efficacy of room temperature onabotulinumtoxinA for glabellar lines treatment compared to freshly reconstituted toxin. All included subjects were informed about the research purposes and voluntarily provided a signed informed consent form to participate in the study. The study followed the Helsinki Declaration.

### 2.2 First Phase: Sterility of Botulinum Toxin at Room Temperature

The sterility test was performed at the Federal University of Paraná, in the Microbiological Quality Control Laboratory, strictly following the protocols described in the Brazilian Pharmacopeia, 7th edition (monograph 5.5.3.2.1—Sterility Test), using the direct inoculation method.

Commercial BoNT‐A vials (Botox, Allergan) from the same batch were reconstituted on day 0 with 1 mL of preservative‐free 0.9% sodium chloride solution. Immediately after reconstitution, the vials were placed in a temperature‐controlled incubator at 37°C and maintained under these conditions for the entire experimental period (60 days). Predefined storage intervals were established: immediate use (time 0), 15, 30, and 60 days post‐reconstitution. At each time point, a vial was removed from the incubator and immediately subjected to sterility testing.

The assays were performed in accordance with the Pharmacopoeia specifications. Fluid Thioglycollate Medium (FTM), incubated at 32.5°C ± 2.5°C, was used for the detection of aerobic and anaerobic bacteria, and Soybean Casein Digest Medium (SCDM), incubated at 22.5°C ± 2.5°C, was used for the detection of fungi and aerobic bacteria. All media were previously qualified through sterility and growth promotion tests. The media were prepared 7 days before the experiment and stored in 10 mL sterile tubes containing 2 mL of medium. For each test, 0.2 mL of the BoNT‐A solution was aseptically inoculated into both FTM and SCDM, ensuring the inoculated volume did not exceed 10% of the medium volume, as required by the Brazilian Pharmacopeia. All manipulations were performed inside a Class II biological safety cabinet in a classified cleanroom environment, with prior UV sterilization of materials for 15 min. Negative controls (uninoculated media) were included for both FTM and SCDM. Additionally, environmental monitoring was performed by exposing Sabouraud Dextrose Agar and Trypticase Soy Agar (TSA) plates inside the biosafety cabinet during the inoculation procedure. All inoculated cultures were incubated under the specified conditions for at least 14 days, with observations at days 7 and 14 for macroscopic evidence of turbidity or microbial growth. The absence of growth in all test media at the end of the incubation period was interpreted as compliance with sterility requirements, in accordance with the Brazilian Pharmacopeia criteria.

### 2.3 Second Phase: Efficacy of BoNT‐A at Room Temperature for Glabellar Lines Treatment

#### 2.3.1 Participants

The sample was obtained from Brazilian individuals with no sex distinction between 25 and 60 years old, complaining about glabellar dynamic wrinkles, presenting moderate or severe forehead and glabellar dynamic lines at severity levels II‐IV according to the Merz 5‐point scale [13]. Exclusion criteria encompassed individuals who received BoNT‐A injections in any region of the face previously. Also, individuals with autoimmune and neuromuscular diseases, who received the antitetanic vaccine or any chemical peels in the face in the previous 6 months to the study initiation, or who underwent any aesthetic procedure in the forehead and glabellar regions during the previous 12 months, and who were currently using drugs that act on neuromuscular junctions.

#### 2.3.2 Randomization and Blinding

Randomization of the individuals was performed by a technician not involved in any other aspect of the study, using an automated software (http://www.randomization.com/) in blocks of four patients. The block size and treatment assignments were unknown to the investigator administering the substances, the investigator evaluating the patients, and the patients themselves. The assigned treatment was placed in a sealed dark envelope and was opened immediately before the injections. Then, the included participants were randomized into two groups: G1 (*n* = 30), participants who received immediately reconstituted BoNT‐A (at room temperature), and G2 (*n* = 30), participants who received a 1‐week stored BoNT‐A, maintained at room temperature.

#### 2.3.3 Treatment

BoNT‐A (100 U, Botox, Allergan, Irvine, CA, USA) was reconstituted with 1.0 mL of preservative‐free saline solution 0.9%, so that every 0.1 mL represented 10 U of the product. Reconstituted BoNT‐A was stored at 37°C in a temperature‐controlled incubator for 7 days before injections or was injected immediately after reconstitution, depending on group assignments. The doses applied in the procerus and corrugator supercili muscles followed the Italian consensus for onabotulinumtoxinaA (Botox) injections [14]. Therefore, 6 U were injected into the procerus muscle, and 6 U into the medial head of the corrugator supercili muscle in a single injection point. The injected dose was not adjusted to the individual aesthetic needs since the neuromodulator effects on durability are mainly dose‐related and would influence the results. Injections were performed using a 1‐mL syringe with a 30‐gauge needle. The injection technique did not differ among the study participants and was performed by an experienced researcher in aesthetic injectables who was not involved in any other process of the study.

#### 2.3.4 Outcomes

Subjects were evaluated five times (baseline, 15, 30, 60, and 90 days) throughout the study. Outcomes were assessed at all evaluations by a researcher blinded to group assignment.

##### 2.3.4.1 Muscle's Electromyographic Activity (EMG)

To capture the electromyographic (EMG) signals, an 8‐channel EMG system (Miotool, Biovision, Wehrheim, Germany) was employed under the supervision of a single operator who underwent calibration (Kappa > 0.80). The system's EMG amplifiers operated within a frequency range of 10–700 Hz with a 3 dB level and featured a sampling rate of 3000 samples per second and a resolution of 2.44 μV per bit. Bipolar surface electrodes of infant size (3.2 × 2.8 cm, Ag‐AgCl disks, Covidien LLC, Canada) were affixed to the targeted muscles after skin preparation involving cleansing with 70% alcohol. A reference electrode was placed on the manubrium of the sternum. For the procerus muscle, electrodes were positioned at the cranial end of the nasal bones, while for the corrugator supercilii muscles, electrodes were situated midway along the cranial border of the eyebrow. Participants were instructed to perform specific facial expressions—glabellar frowning (angry upper facial expression)—in front of a mirror to familiarize themselves with their maximum voluntary contraction (MVC) for each expression.

During EMG signal recording of MVC, participants remained seated upright in a chair with head and shoulders relaxed, ensuring the Frankfurt plane was parallel to the floor. They were instructed to contract the designated muscles maximally for 5 s. This protocol was repeated three times with a 1‐min rest period between repetitions to mitigate fatigue. The entire EMG signal was captured at a frequency of 1000 Hz using an analog‐to‐digital conversion acquisition device and recorded with MiotecSuite software (Miotec Equipamentos Biomédicos, Brazil). Subsequently, the EMG signal underwent bandpass filtering (20–500 Hz) to calculate the Root Mean Square (RMS) value representing the 5‐s MVC of the facial muscles. Offline analysis of the MVC data was conducted using MiotecSuite software, with statistical analyses based on the mean values derived from the three recorded trials.

##### 2.3.4.2 Glabellar Lines Severity

The evaluation of glabellar lines in the contracted and rest state adhered to the Merz‐5 point scale. This scale quantifies visible lines across five grades: 0 for absence of lines, 1 for mild lines, 2 for moderate lines, 3 for severe lines, and 4 for very severe lines [15]. A researcher not involved in other study processes conducted these assessments through visual examination of patients during pre‐ and post‐treatment follow‐up sessions.

##### 2.3.4.3 Patient's Perceived Satisfaction with BoNT‐A Treatment

The FACE‐Q scales constitute validated instruments designed to gauge outcome expectations and patient satisfaction, both before and after undergoing treatment. Specifically, the “Face‐Q Appraisal of Lines between eyebrows” scale was employed to assess the degree of concern experienced by participants regarding the area between their eyebrows over the preceding week. This assessment employs a 4‐point scale where responses range from 1 (not at all bothered) to 4 (extremely bothered). Scoring for the FACE‐Q Appraisal of Lines Between Eyebrows scale involves summing the item scores to derive a total raw score. Subsequently, a conversion table tailored for each scale is utilized to transform the raw score into a standardized score ranging from 0 (indicating the poorest outcome) to 100 (indicating the optimal outcome) [16].

The authors have affirmed compliance with the FACE‐Q license agreement, enabling the use of these scales specifically for non‐profit academic research purposes. (Copyright notice: “Copyright 2013 Memorial Sloan Kettering Cancer Center, New York, USA. All rights reserved”).

#### 2.3.5 Data Analysis

Normal distribution of the variables was verified by means of the Kolmogorov–Smirnov test. Differences from the G1 and G2 group within each time point of Merz Scale and FACE‐Q appraisal of lines between eyebrows, which lacked normal distribution, were evaluated by means of the nonparametric Mann–Whitney U‐test. The Friedman test was performed to compare data within each group along time points for these variables. Two‐way repeated‐measures analysis of variance (ANOVA), a parametric test, was applied to electromyographic activity data to verify whether there were statistically significant differences between the treatment protocols (factor 1) and time points (factor 2). The means were compared by the Bonferroni test for post hoc comparison. A significance level of *p* < 0.05 was used for all tests. All statistical analysis was performed using statistical software (IBM SPSS Statistics, v21.0; IBM Corp.).

## 3 Results

### 3.1 First Phase: Sterility of BoNT‐A

Microbiological analyses demonstrated that both Fluid Thioglycollate Medium and Soybean Casein Digest Medium remained clear and free of turbidity throughout the incubation period, as assessed by visual inspection on days 7 and 14. This result indicates the absence of bacterial or fungal contamination in all storage conditions evaluated.

### 3.2 Second Phase: Efficacy of BoNT‐A for Glabellar Lines

#### 3.2.1 Electromyography Activity

The evaluation of the electrical activity of procerus and corrugator muscles exhibited significant differences only for the time factor [*F* (1.58; 45.72) = 111.82; *p* < 0.001]. For treatment [*F* (1.00; 29.00) = 0.13; *p* = 7.20] and the interaction among factors [*F* (1.62; 46.95) = 166.07; *p* = 0.69], the differences were not statistically significant. The electrical activity at different time periods (15, 30, and 60 days) was significantly lower compared with the baseline values, in both groups, although it had a slight but statistically significant increase after 90 days (Table 1) also in both groups.

**TABLE 1:** Electromyographic activity (mV) of the procerus and corrugator muscles at different time points before (baseline) and after treatment protocol.

	G1	G2
Baseline	51.62 (±23.53) Aa	48.71 (±19.18) Aa
15 days	26.37 (±11.75) Ab	24.98 (±11.65) Ab
30 days	26.06 (±10.56) Ab	26.44 (±12.08) Ab
60 days	26.62 (±10.21) Ab	25.75 (±10.61) Ab
90 days	29.10 (±9.74) Ac	29.40 (±10.66) Ac

*Note:* Data are shown as the mean ± SD. Different capital letters show statistically significant differences (*p* < 0.05) between treatments protocols within each time point. Different lowercase letters show statistically significant differences (*p* < 0.05) among the time points within each treatment protocol.

#### 3.2.2 Glabellar Lines Severity

Statistical analysis revealed significant differences in the Merz Scale (Table 2) between groups (*p* < 0.05) for most assessed time points, except at 90 days. In general, G2 had higher values on the scale compared to G1. Within each group, the time factor also showed an effect for G1 [*X* ^2^ [4] = 100.65; *p* < 0.001] and G2 [*X* ^2^ [4] = 98.99; *p* < 0.001]. Within each treatment protocol, there was at least a 1‐point improvement compared with baseline, which was maintained until 60 days.

**TABLE 2:** Merz Scale (0–5) at different time points before (baseline) and after treatment protocol.

	G1	G2
Baseline	2.0 (1.0) Aa	3.0 (1.0) Ba
15 days	1.0 (2.0) Ab	2.0 (1.0) Bb
30 days	1.0 (1.0) Ab	2.0 (1.0) Bb
60 days	1.0 (1.0) Ab	2.0 (1.0) Bb
90 days	2.0 (0.25) Aa	2.0 (0.25) Ab

*Note:* Data are shown as the median (interquartile range). Different capital letters show statistically significant differences (*p* < 0.05) between treatments protocols. Different lowercase letters show statistically significant differences (*p* < 0.05) among the time points within each treatment protocol.

#### 3.2.3 Patient Satisfaction With the Treatment

Table 3 reports the FACE‐Q appraisal of lines between eyebrows scores. Patients from G2 presented significantly higher satisfaction with the treatment compared with G1 just at the 15‐ and 30‐day follow‐up (*p* < 0.01). In addition, the time factor had a positive influence on both G1 [X^2^ [4] = 94.09; *p* < 0.001] and G2 [X^2^ [4] = 60.54; *p* < 0.001] groups, showing a significant improvement in patients' satisfaction with the treatment when comparing baseline scores with all follow‐ups. However, after 30 days of treatment injections, there were not significant differences (*p* < 0.05) between time points in both groups.

**TABLE 3:** FACE‐Q appraisal of lines between eyebrows at different time points before (baseline) and after treatment protocol.

	G1	G2
Baseline	45.0 (20.0) Aa	55.0 (19.0) Ba
15 days	52.0 (14.0) Ab	59.0 (8.0) Bab
30 days	59.0 (8.0) Ac	63.0 (9.0) Bbc
60 days	68.0 (18.0) Ac	68.0 (18.0) Ac
90 days	62.1 (±6.5) Ac	61.9 (±6.3) Abc

*Note:* Data are shown as the median (interquartile range). Different capital letters show statistically significant differences (*p* < 0.05) between treatments protocols within each time point. Different lowercase letters show statistically significant differences (*p* < 0.05) among the time points within each treatment protocol.

## 4 Discussion

The stability and clinical performance of BoNT‐A have long been topics of interest, with previous investigations exploring variables such as cold storage conditions, the interval between reconstitution and administration, and the potential for contamination when the product is not used immediately. Existing evidence generally suggests that these factors exert minimal influence on BoNT‐A's efficacy [9]. Nevertheless, to the best of our knowledge, no prior study has examined the aesthetic outcomes and sterility of BoNT‐A reconstituted and stored at higher temperatures. The present findings challenge conventional assumptions by demonstrating that exposure to elevated temperatures (37°C) and prolonged storage durations do not compromise BoNT‐A's aesthetic effectiveness or sterility. These results expand the current understanding of BoNT‐A handling protocols and may have practical implications for clinical workflows and product utilization.

The consensus regarding the storage and reuse of previously reconstituted neuromodulators indicates that BoNT‐A, when reconstituted under clinically appropriate conditions, can be stored using different cold storage protocols for up to 4 weeks without significant risk of contamination or loss of efficacy [6]. Clinical studies on onabotulinumtoxinA have similarly reported that refrigerated or frozen storage for up to 14 weeks does not result in bacterial or fungal growth [9, 17, 18, 19]. These findings are noteworthy given that the current manufacturer guidelines recommend storage between 2°C and 8°C after reconstitution, with use within 24 h, to minimize the risk of microbial proliferation and preserve potency.

Our findings partially align with this literature, as we observed that the sterility of reconstituted onabotulinumtoxinA was maintained even after 8 weeks of storage at 37°C. The complete absence of microbial contamination under these conditions suggests that, when aseptic reconstitution and handling protocols are rigorously followed, the risk of microbial proliferation may be considerably lower than anticipated, even at temperatures well above recommended ranges. However, it is important to note that all experimental procedures in this study were conducted in a sterile laboratory environment. In contrast, real‐world clinical settings often lack such stringent sterilization controls, raising legitimate concerns about potential contamination risks during routine handling and storage in outpatient facilities.

The 0.9% sodium chloride solution used for BoNT‐A reconstitution in this study was pharmaceutical‐grade sterile water for injection, isotonic, and free of preservatives or antimicrobial agents. Thus, microbial safety was ensured not by any intrinsic antimicrobial effect of the diluent, but by strict aseptic techniques during reconstitution and storage. In clinical settings, contamination risk primarily arises from breaches in aseptic barriers, such as contact with non‐sterile needles, repeated punctures, or stopper removal. Although stopper removal may minimize wastage, recovering up to 5 units per vial, this practice is strongly discouraged as it compromises sterility and increases contamination risk [20].

In addition to the preserved aesthetic outcomes, it is important to emphasize that no significant differences were observed in the electromyographic parameters between the experimental and control groups. This finding suggests that the functional muscular response remained stable regardless of storage conditions, reinforcing the reliability of the observed clinical results. Notably, unlike previous investigations that focused primarily on aesthetic evaluation or microbiological safety, the present study incorporated electromyographic analysis as an objective and quantitative tool to assess muscle activity. This methodological approach represents an advance in the field, as it provides complementary evidence that supports the efficacy of BoNT‐A under the tested conditions.

It is noteworthy that the results from the Merz scale and FACE‐Q provided complementary perspectives when compared with the EMG findings. While the Merz scale indicated slightly better outcomes for Group 1, patient‐reported satisfaction through FACE‐Q was higher in Group 2, particularly in the first month. This apparent discrepancy may be explained by the fact that participants in Group 2 had more severe glabellar wrinkles at baseline, which likely enhanced their subjective perception of improvement. Importantly, the main driver of differences across all analyses was time, with significant improvements observed after treatment application, supporting the effectiveness of onabotulinumtoxinA stored at room temperature.

Overall, our findings challenge the long‐standing assumption that BoNT‐A must be stored exclusively at low temperatures to maintain its efficacy and sterility. By demonstrating that BoNT‐A reconstituted and stored at elevated temperatures (37°C) for extended periods retains both aesthetic performance and microbial safety under controlled conditions, this study broadens the evidence base for more flexible storage protocols. While caution is warranted when extrapolating these results to real‐world clinical environments, where sterility standards may vary, the data presented here open new avenues for optimizing BoNT‐A handling, reducing waste, and potentially improving treatment accessibility. Future large‐scale, multicenter studies are warranted to confirm these results across diverse settings and to refine guidelines that balance safety, efficacy, and practicality.

### 4.1 Limitations and Strengths

Although our findings did not reveal a negative impact of reconstitution time or temperature on the duration of the aesthetic effects of BoNT‐A, caution is warranted when interpreting these results due to certain limitations. Specifically, this study did not assess the potential accumulation of other contaminants in reconstituted BoNT‐A vials, such as additional toxins or viruses. Moreover, sterility testing was performed through turbidity analysis, as described in the pharmacopeia, which is a subjective method and may not fully capture contamination risks.

Future studies are encouraged to employ more sensitive and objective approaches to verify microbial contamination. Furthermore, individualized dosing was not implemented, which may have influenced outcomes in patients requiring higher doses to achieve optimal aesthetic results. Nevertheless, all patients exhibited significant aesthetic improvement and reported high satisfaction with the treatment. Importantly, the combination of subjective and objective measures enhances the reliability of our findings.

## 5 Conclusion

It can be concluded that onabotulinumtoxinA stored at higher temperatures, even for longer periods, maintained both its aesthetic efficacy and sterility under clinical and laboratory conditions, respectively.

## Author Contributions

Conceptualization: Ruth Yimna Del Socorro Blanco Rodriguez and Mariana Barbosa Câmara‐Souza; Methodology: Ruth Yimna Del Socorro Blanco Rodriguez, César Nunes Giracca, Fábio Seigi Murakami, Giancarlo De La Torre Canales, Thaís Caroline Schwartz Saldanha Vaz and Mariana Barbosa Câmara‐Souza; Formal analysis and investigation: Ruth Yimna Del Socorro Blanco Rodriguez, Gislaine Paulovski, Lucas Alexandre de Sousa Mendes and Thaís Caroline Schwartz Saldanha Vaz and Fábio Seigi Murakami; Writing – original draft preparation: Giancarlo De La Torre Canales, Fábio Seigi Murakami, Jairo Matozinho Cordeiro, and Mariana Barbosa Câmara‐Souza; Writing – review and editing: Giancarlo De La Torre Canales, Jairo Matozinho Cordeiro, and Mariana Barbosa Câmara‐Souza; Resources: Giancarlo De La Torre Canales, Fábio Seigi Murakami and Mariana Barbosa Câmara‐Souza; Supervision: Giancarlo De La Torre Canales, Fábio Seigi Murakami and Mariana Barbosa Câmara‐Souza.

## Ethics Statement

The randomized controlled single‐blinded phase of this study was approved by the Research Ethics Committee of Uningá University, Paraná, Brazil (CAAE #59261322.0.0000.5220). All included subjects were informed about the research purposes and voluntarily provided a signed informed consent form to participate in the study. The study followed the Helsinki Declaration.

## Conflicts of Interest

The authors declare no conflicts of interest.

## Data Availability

The datasets generated during and/or analyzed during the current study are available from the corresponding author on reasonable request.
